# Drama as a pedagogical tool for practicing death notification-experiences from Swedish medical students

**DOI:** 10.1186/1472-6920-11-74

**Published:** 2011-09-28

**Authors:** Anna Nordström, Anncristine Fjellman-Wiklund, Tomas Grysell

**Affiliations:** 1 Department of Surgical and Perioperative Science, Sports Medicine, Umeå University, S-901 85 Umeå, Sweden; 2 Department of Community Medicine and Rehabilitation, Rehabilitation Medicine, Umeå University, S-901 87 Umeå, Sweden; 3 Department of Community Medicine and Rehabilitation, Physiotherapy, Umeå University, S-901 87 Umeå, Sweden; 4 Division for Development of Teaching and Learning, Uppsala University, S-751 20 Uppsala, Sweden

## Abstract

**Background:**

One of the toughest tasks in any profession is the deliverance of death notification. Marathon Death is an exercise conducted during the fourth year of medical school in northern Sweden to prepare students for this responsibility. The exercise is designed to enable students to gain insight into the emotional and formal procedure of delivering death notifications. The exercise is inspired by Augusto Boal's work around Forum Theatre and is analyzed using video playback. The aim of the study was to explore reflections, attitudes and ideas toward training in delivering death notifications among medical students who participate in the Marathon Death exercise based on forum play.

**Methods:**

After participation in the Marathon Death exercise, students completed semi-structured interviews. The transcribed interviews were analyzed using the principles of qualitative content analysis including a deductive content analysis approach with a structured matrix based on Bloom's taxonomy domains.

**Results:**

The Marathon Death exercise was perceived as emotionally loaded, realistic and valuable for the future professional role as a physician. The deliverance of a death notification to the next of kin that a loved one has died was perceived as difficult. The exercise conjured emotions such as positive expectations and sheer anxiety. Students perceived participation in the exercise as an important learning experience, discovering that they had the capacity to manage such a difficult situation. The feedback from the video playback of the exercise and the feedback from fellow students and teachers enhanced the learning experience.

**Conclusions:**

The exercise, Marathon Death, based on forum play with video playback is a useful pedagogical tool that enables students to practice delivering death notification. The ability to practice under realistic conditions contributes to reinforce students in preparation for their future professional role.

## Background

Delivering a death notification is an emotionally difficult task regardless of previous experience. There are no words that can lessen the impact of the horrendous message. Every year millions of people are notified that a close relative has died. The manner in which the news is communicated may be crucial to the emotional recovery of relatives. The management of both patients and their relatives is of vital importance when one is suffering from a severe disease [1]. The emotional state of both patients and their relatives can elicit emotions in health care staff which can lead to hampered communication [2]. It is crucial for health care workers to observe and interpret these emotions in order to understand the emotions that arise within [3-7].

Forum Theatre, originally developed by Augusto Boal [8-10] incorporates audience participation into a performance such that audience members make suggestions to actors in order to alter the outcome of a scene. Engaging in a performance can incite questions, experiences and issues that may otherwise be overlooked. In a broad meaning forum play is a simulation education because it is a simulation of a specific event with a specific goal. Forum play may be a useful exercise in socio-analytic role playing in the teaching of delivery of death notification. We developed a unique method termed "Marathon Death" to illustrate and solve problems in human relations based on Forum Theatre. We have successfully used this method for preparing medical students for the delivery of death notifications. In the model, all students are engaged in a form of Forum Theatre but with a focus on a pedagogic situation which we called forum play. Marathon Death is a valuable tool for discussing difficult questions and issues pertaining to death notification, developed specifically for use as a training instrument for action and understanding of human behaviour at the Medical school at Umeå University, in northern Sweden. Through the process, teachers become actively engaged with students, developing strong relationships and trust.

During the fourth year of medical school students participate in a three-day retreat as part of a course in professional development. The course addresses challenging patient-physician communication issues and the students are not informed of specific exercises within the course curriculum in advance. In groups of 8-10, students have the opportunity to participate in an exercise on the communication of a death notification. All groups work simultaneously and each group is led by a teacher. The teacher plays the role of next of kin and gives a realistic introduction to the exercise by describing the events leading up to the death of his/her husband/wife as realistically and vividly as possible. The objective of the exercise is for the medical student to take on the role of medical doctors and relay the death notification to the next of kin without paraphrasing. The element of surprise is an important component for realism. After initial information about the exercise, students wait outside the classroom until their turn. The exercise is performed without an audience and is videotaped. Students are told that the exercise will start while the relative is in the waiting room anticipating news of their spouse. The teacher, playing role of next of kin, pretends to be unaware that his or her spouse has died and inquires as to the state of the relative. The exercise is stopped when the student utters the word 'died' or 'deceased'. The entire interaction takes place over a few minutes. Many students become emotionally affected by the experience, sometimes to the extent that they have difficulty perceiving when the teacher ends the exercise and goes out of character. Each student then has the opportunity to privately reflect on their feelings while their colleagues participate in the exercise. After all students in the group have taken part in the exercise the group gathers for discussion and reflection. Subsequently, each taped exercise is analyzed. During playback the recording is paused and participants are able to re-enact a continuation on the scene in our special model of forum playing. The novelty in the way we use forum play lays in the use of co actors, facilitators and play back that enhances the active learning through interaction and dialogue. Through the facilitator, students are helped to identify personal learning objectives and to practice behaviours through interaction with colleagues. The facilitator also draws feedback from peer observers during the playback moment enabling an added understanding from the work context.

The aim of the study was to explore reflections, attitudes and ideas toward training practices in the deliverance of death notification among medical students.

## Methods

### Study design and participants

A qualitative approach was used in the exploration of student attitudes and ideas toward the training exercise. Interviews with semi-structured questions promoting open ended content and direction were conducted. The interviews were analyzed through qualitative content analysis [11]; a method of analyzing written or verbal communication in a systematic way [12]. The method was selected based on its usefulness in the analyses of experiences, reflections, and attitudes [13].

A maximum variation sample was chosen with the following variables: males and females, large age range, variety of nationalities, variety of prior experience in death notifications and participation in Marathon Death exercise groups. During the fall of 2008, 85 students were involved in the Marathon Death exercise. Of the 85 students who participated, 10 were asked to volunteer in the study. All 10 students agreed to participate in the interviews that took place within two weeks after the exercise. Pertinent background information of each student is given in Table 1.

**Table 1:** Participant background information

**Emilia **35 years, from Sweden, had never been exposed to a death notification neither professionally nor private.
**Erik **20 years of age, from Sweden, His experience from death notification was limited. He had received one notification by telephone; it was when his brother died unexpected.
**Fredrik **25 years, from Germany. He had received a death notification when a relative died when he was younger, at that time his father had informed him.
**Kajsa **25 years, from Sweden. She experienced the trauma of having her father died unexpectedly under tragic circumstances after a longer illness and she got the notification over the telephone of the care taking staff. Kajsa usually put herself or her family in the situations she encounters during her education. When she faces a patient she always see a relative in the patient and think of how she would feel if a physician came and gave the notification that a close relative of hers had died. The thought is always with her in clinical situations.
**Li **25 years, from China, had experienced a death notification through a letter when a relative had died.
**Lina **25 years, from Sweden, had never been exposed to a death notification neither professionally nor private.
**Lotta **35 years, from Sweden. She had never had the experience of a death notification but had gotten a notification several years ago regarding a distant relative.
**Peder **20 years, from Sweden, A close relative to Peder died a few years ago and it was the parents who conveyed it over the telephone. The notification was unexpected although the relative was elderly. Peder discovered that it was rather nice at that time to get a moment to himself and just reflect over the news.
**Peter **20 years, from Sweden. Peter had a close relative who died unexpected and at that time it was his father who gave the notification. He also had had other relatives who had died after a period of disease. In those cases he thought that it was more of a relief for the relatives when they finally died. The horrendous of the situation was the disease and all that came before death.
**Sara **20 years, from Sweden, had experienced two deaths in the family. She had been there with her grandmother when she died expectedly after experienced a good, long life. Her grandmother had gotten weaker and weaker and it had been a relief when it came. It had felt nice when all family members had been able to say their farewell.

Participant background information pertinent to the Marathon Death exercise.

The study design and protocol were approved by the head of the Professional Development Course faculty at Umeå University, Sweden. The background and aim of the study was explained to all students before participation. The work was carried out in accordance with the Declaration of Helsinki, including, but not limited to there being no potential harm to participants. The students were assured of confidentiality and strict anonymity. Informed consent of students was obtained for publication.

### Collection and analysis of data

Data were collected from semi-structured interviews to capture participant experiences from a personal perspective [14]. The interview was guided by the following topics: student experience of the death notification exercise including the establishment of contact with the next of kin and the student's own perception of their emotions (i.e. interest, warmth and empathy) in that situation; the amount of information the next of kin perceived, use of time during the exercise and the student's interpretations of the relative's feelings when the death notification was delivered. The interview guide was used throughout the study. Each interview was performed by one researcher and lasted about 60 minutes. The interviews were tape-recorded with permission from the students and then transcribed verbatim.

The interviews were analyzed by means of qualitative content analysis performed in several steps [11], using a deductive content analysis approach with a structured matrix [15]. Bloom's taxonomy [16] was used as the structured matrix. The data were analyzed by a teacher (who is also a physician) of the professional development course, a pedagogue experienced in forum play and qualitative research and a physiotherapist with experience in occupational health care and qualitative research. Each author performed an individual preliminary analysis of the entire text in relation to the aims of the study. The text was divided into meaning units, each comprised of several words, sentences, or paragraphs containing interrelated aspects of content and context. Reoccurring, but independent words and phrases regarding thoughts, emotions and attitudes towards the exercise emerged. Taking context into consideration, the units were condensed and each was labelled with a code related to the three levels of Bloom's taxonomy (the cognitive, affective and psychomotor domains) (See Table 2). An additional rereading of the transcripts was performed with the aim of further analyzing ways in which these categories were linked. Excerpts pertaining to identified semantic relationships between specific expressions were clustered to form thematic relationships, which in turn were analyzed for linked thematic patterns. Emerging categories and thematic patterns were synthesized and analyzed based on Bloom's taxonomy [16].

**Table 2:** Examples of codes and categories

Codes	Categories according to Bloom's taxonomy
*Professionalism*	Cognitive Domain

*-expressions of interest, listening, planning ahead, expectations, content of communication, how to communicate, how to react*	

*The need for time* *A soothing environment*	

*Unpleasant* *Focused* *Excitement* *Low-spirited* *Relieved* *Content* *Self-reliance* *Sensitivity* *Revised behaviour*	Affective Domain

*Body language* *Body contact* *Eye contact*	Psychomotor Domain

Codes and categories describing experiences during the Marathon Death training exercise according to Bloom's taxonomy.

## Results

Analysis of the reflections, attitudes and ideas towards training in delivering death notifications among the medical students are presented as both codes and descriptive quotations (in italic) sorted into categories according to Bloom's taxonomy (Table 2).

### The cognitive domain

Reflections on the learning outcome of the exercise were categorized according to **the cognitive domain **of Bloom's taxonomy. Overall, a high degree of comprehension and application of knowledge was reported. Participants reported that conversations were not as anticipated. Students expected a calmer situation where the next of kin would react as they imagined people in real-life should react in the same situation. The exercise created a virtual situation where students described reflecting over their own and the next of kin's reactions and emotions and their own future as a professional physician. Participants began to understand that there is no set of rules for human behaviour in this special kind of situation. Some students presented an analysis on what they could have done differently and pondered over possible alternate outcomes; *I have experienced something that will *occur *in the future, I could very well *be *the next of kin in real life and I could also be the one informing someone of the death notification*.

The students expressed the importance of *being professional *when giving notifications. Being professional included *expressing interest *while *simultaneously formulating a response. Control *of the situation was perceived as vital including *having a plan *along with having a *period of the time *before the notification in a *soothing environment *in order *to get oneself together before *delivering the news. *How to convey the message *was frequently discussed; *how much information to give*, *what to say, what not to say, how to express death (e.g. deceased, died...) *and how to *end the conversation. If I should start with describing the events leading up to the death of his or her relative or if I should cut right to the core saying that the relative was dead...//*. How to convey the message also included *setting the tone*; *how smoothly you can make it through the situation without giving (the notification) abruptly...*// *how to set the tone to make the next of kin realize from the start that something was not right. It would be wrong to start with a happy tone only to have to later arrive at the dreaded notification*. The students tried to imagine *what to expect *during the notification and *how to react *towards the reaction from the next of kin and some students decided to improvise as the situation progressed; *She (next of kin) was talking all the time. I didn't know if I was to interrupt her or wait until she stopped talking and became quiet*.

### The affective domain

The emotional aspects of the exercise were investigated using the **affective domain **of Bloom's taxonomy. All students experienced *a tension *prior to the exercise and great *anticipation*. Some participants had positive expectations such as *excitement *to try to give a death notification and a bit of *an adrenaline kick *to *try to handle *the situation. Others were *relieved *since they had *been performing another exercise earlier that had turned out well the day before*. Yet, others perceived the exercise as *difficult *and felt *nervous *and *insecure*. There were feelings of *unpleasantness *and some students were *worried *how the experience would affect their emotions during the exercise and how they would react in the future, in their professional life.

The forum play was perceived as *artificial yet at the same time very real*. Some felt *focused *and *at ease *when the exercise started. After the exercise all students were somehow *affected*. Some participants felt *upset, shocked *and *stressed*. There were feelings of *inadequacy; I realize how incredibly inadequate I am. Normally I feel that I usually can talk my way through most things and know fairly well what to say but I just had a blackout... it was really unfamiliar*. At the same time the students were *content *with their *gained insight *but, *it was rather heavy*.

### The psychomotor domain

**The psychomotor domain **of Bloom's taxonomy was used to express manual and physical skills. Most students reflected on their *body language *and on *physical body contact *with the next of kin and how that started the dialogue; *I took his *(next of kin) *hands and I also experienced that he searched for *mine. *I felt that I could ask questions, that we had good communication*. *I had a lot of body contact, I could see on the video afterwards. It was nothing I thought about but it was what I tried to do, partly when I took her around the shoulders, and then later when she was upset talking about the upcoming dinner *(plans that she had made), *then I took rather firmly her hand with both *(of my) *hands and said; "your friend will have to wait" and looked her once again in the eyes to subdue her chatter*.

The importance *of portraying the same *(emotion) *in both speech and in body language *while delivering information to the next of kin realize from the start was discussed; *When she *(next of kin) *started to chatter hysterically I didn't listen and lost focus, I was rather upright in my body language and I took hold of her shoulders. When she walked around I took her around her shoulders and looked her rather determined in the eyes and said; "Now, I want us to sit down." And then she *sat *right away*. Having good *eye contact *with the next of kin when giving the notification was also important; *...to get eye contact, to make sure we were on the same level*.

### The learning process

The following is the reflections of one participant (Fredrik) on the learning process of the exercise.

I had to decide If I would start with describing the course of events to the next of kin leading up to the death of the patient, or if I would go straight to the punch line; that the patient had died, or if I would start to unravel...// I started to think how I could set the tone from the start so that the next of kin receiving the information would understand from the beginning that all was not right. It would have felt wrong to have a merry tone at the start only to later come to the final information; "well your husband is dead." There would be no smooth transition, so to speak, regarding the information/While there is no way to make a true smooth transition in a situation like that, it is important not to be abrupt. It was important to decide whether to talk about what had happened and what went wrong and so on, in which case the next of kin may say; "well...is he or she alive or not?" I feel that for myself I wouldn't want to sit and listen to someone talking about cardiac arrest and resuscitation attempts for very long, but would rather pop the question if I didn't understand what had happened...

*After the exercise I thought it is good just to be put in this situation, to be able to prepare for it. If someone told you; "you must notify someone about the death of their relative." You would start to process, how should I go about this? What strategy should I use? What can I expect will happen... right there and then? A lot is won just by the thought process. If instead you came to a room were someone would say; "the next of kin has already been notified", then you still have done your work and by that learned a whole lot. The...video recording was actually really good for the exercise. I was able to observe that I tried to connect with the person I spoke to, what I said, that I tried to take hold of them, to get eye contact, to make sure we were on the same level. I attempted at least to form some communication scheme...to get the chance to give my information. It was at least an honest attempt, although I thought that I didn't succeed with it. In the aftermath I think that you should prepare for the unexpected (pertaining to) reactions from the next of kin and you shouldn't take for granted that the next of kin will act in a certain way. You cannot expect that the next of kin will sit quietly and calmly waiting to be served the news, They may bombard you with information and questions before you get a chance to gather your thoughts on what to say. You need to have a plan B the whole time*.

The learning process becomes evident when using Bloom's taxonomy to analyze the thoughts of the students. As exemplified in the above reflections there is a clear progression in learning. Prior to the exercise Fredrik portrayed *motivation*: he demonstrated *sensitivity *towards the individual he was about to meet and had formulated a plan on how to carry out the exercise. After the exercise he reflected over the need for balance; comparing, relating and synthesizing values. He exhibited *self-reliance *and thought of the possibilities of how to *revise his behaviour *in light of the experience. These concepts may be interpreted as components of learning in the affective domain of Bloom's taxonomy; responding to and evaluating an experience. Fredrik reflected over the data and gave an interpretation of *how he would carry out the task *he was given. After the exercise he portrayed the ability to analyze the exercise and the learning outcome. He gave a synthesis of the exercise and what to *improve *on. These reflections can be construed as developments in intellectual skills (i.e. the cognitive domain of Bloom's taxonomy).

## Discussion

The exercise, Marathon Death, was introduced into the curricula at the medical school at Umeå University, Sweden to meet the need for training on communication in difficult situations. Marathon Death is a useful pedagogic exercise and model, based on Forum Theatre that enables students to train on giving death notifications. The exercise was perceived as a valued training experience in a safe and controlled environment. In addition, students and teachers developed positive relationships and trust through the process. The students' thoughts and feelings prior to the exercise varied and did not seem to reflect gender, age, nationality, or prior experience of death notification. The students expressed the importance of having an opportunity to train before they faced this difficult task in their future professional lives. The ability of a physician to communicate death notifications to relatives in an appropriate manner can have positive effects on the grief response and subsequent resolution of the loss [17]. Despite the importance of this process, little formal training is given to health care staff during their education or in their professional life [4,18-22].

Attitudes, experiences and emotions are important complex learning outcomes. It is important to investigate all aspects of the learning process in the evaluation of an exercise like Marathon Death. The current study used Bloom's taxonomy [16] to categorize reflections made by students during interviews. The cognitive domain explored knowledge and the development of intellectual skills. The affective domain investigated the emotional reaction of the students to the task. The psychomotor domain included reflections on body language and eye contact during the exercise but was not analyzed to any higher extent.

The attitudes elicited by the exercise were investigated. The exercise was specifically designed to bring out emotions and reflections. According to Bolton and Heathcote the performance enables the active (rather than passive) attainment of knowledge in a situation [23]. Thus, the design of the exercise contributes an additional dimension of education; the students are provided the opportunity to use the knowledge and skills that are important for their profession. Rather than reconstructing and reproducing knowledge, the students become active players in their own education; they are given the chance to put their newly acquired knowledge and skills to use. The affective domain of Bloom's taxonomy [16] was used to explore the emotional responses that students expressed. All students were affected by the exercise instructions even prior to entering into the forum play. It is clear that the exercise starts an immediate emotional process even while the students reflect over the exercise instructions.

The psychomotor domain of Bloom's taxonomy [16] was not the main focus of the Marathon Death exercise; focus was instead on the cognitive and affective domains. It is difficult to evaluate the psychomotor domain through conversational skills. However, most of the students reflected on the body language they observed in the videos which could be interpreted as an early stage in the learning of complex skills.

The results of the current study indicate that Marathon Death is a valuable tool for the training of medical students in delivering death notifications. Several of the students chose to give the notification in such a way that they felt that they, themselves would prefer to receive a death notification. The process of reflecting on their own preferences if the roles were reversed may have enhanced the emotional component of the exercise. It is of great value for the students to have insight into how they may act under pressure; how they may express themselves and how they may treat individuals in crisis while at the same time dealing with their own feelings. However, the exercise comes with risk. The perception of failure in such an exercise may increase tension and make job demands feel insurmountable. While the exercise comes with risk, it is clear that the feedback from peers and an experienced tutor fulfil an important function, pointing towards positive features and thereby strengthening self-confidence. Moreover, the recordings provide the opportunity to pause at any point during the interaction and then to continue to act out the rest of the scene in front of the group, enhancing learning by dividing the exercise into meaningful components. The great variety in experience and situations of each participant made the leadership role difficult for the teachers. Thus, experience and support for the teachers in the form of meetings prior to and following the exercise is essential. In addition, it is recommended that some form of support for students is provided in the event that the exercise evokes strong feelings that need some extra assistance to work through.

Students with a prior negative experience involving a death notification in their own private life were more likely to report experiencing moderate to very high stress during the exercise. These participants reflected over their private experiences and their desire to notify the next of kin in the exercise according to how they themselves would have preferred receiving a death notification. Interestingly, there were no spontaneous comments from the students of perceived stress experienced either prior to or during the exercise on the perceived usefulness of the exercise. In other words, even students who perceived stress also perceived the task as useful.

Interpretation of the results is based on qualitative data. Shenton describes that qualitative data are specific to the context and the individuals investigated and that care must be taken when interpreting results [24]. The participants in the present study are medical students and caution must be used when generalizing results to any other population. It may be argued, however that there is a certain amount of transferability of these qualitative findings to students in contexts similar to those in the present study. An advantage of the study design is the detail with which the data were described and the use of Bloom's taxonomy [16] for analysis of this special kind of pedagogical data. The study design can be repeated in a variety of contexts to explore whether students in similar (and different) contexts learn in similar ways [24,25]. Such findings will extend the existing body of knowledge with regard to student learning.

There is a symbiotic relationship between information garnered from qualitative and quantitative data. Qualitative research is effective in the development of hypotheses in "newly emerged or under-researched areas" which can then be tested more effectively using quantitative measures at a later stage [25]. Further quantitative studies are needed to investigate other aspects of student learning.

## Conclusions

The current study offers intriguing preliminary findings concerning a new valuable exercise in death notification training. Marathon Death is an effective method of encouraging self-reflection concerning the process of doctoring and the doctor-patient relationship in the process of death notification. Marathon Death appears to be a valid exercise for students in reducing the anxiety and stress inherent to death notification. Overall, forum play with a playback feature should be considered a new form of simulated learning and a useful method of enhancing and enriching training in death notification.

## Competing interests

The authors declare that they have no competing interests.

## Authors' contributions

AN conceptualised and implemented the study, developed study materials, collected and analysed data, and drafted the manuscript, AFW analysed and interpreted the data and drafted the manuscript, TG conceptualised the study and supervised data collection. AN and AFW helped review drafts of the manuscript. All authors have read and approved the final manuscript.

## Pre-publication history

The pre-publication history for this paper can be accessed here:

http://www.biomedcentral.com/1472-6920/11/74/prepub
